# Exclusion From Childcare Centre Attendance in Toddler and Preschool Patients in a Psychiatric Day Clinic: Patterns From Comparison of a 2018/2019 and a 2023/2024 Cohort

**DOI:** 10.1111/cch.70330

**Published:** 2026-08-14

**Authors:** Linda Meredith Bonnekoh, Lennart Friese, Ruth Helena Fellmeth, Héctor Andrés Sánchez Guerrero, Selina Hansen, Jörg Michael Müller, Julia Hubbert, Udo Dannlowski, Georg Romer, Marius Janßen

**Affiliations:** ^1^ Department of Child and Adolescent Psychiatry, Psychosomatics and Psychotherapy, University Clinic Münster University of Münster Münster Germany; ^2^ Institute for Translational Psychiatry University of Münster Münster Germany; ^3^ LWL‐University Hospital for Child and Adolescent Psychiatry Ruhr‐University Bochum Hamm Germany; ^4^ Institute for Biomagnetism and Biosignalanalysis University of Münster Münster Germany; ^5^ Department of Psychiatry, Protestant Hospital of the Bethel Foundation, Medical School and University Medical Center OWL Bielefeld University Bielefeld Germany

**Keywords:** behavioural disorders, childcare, childcare centres, exclusion, externalising disorders, preschool, toddler

## Abstract

**Background:**

Childcare centres for toddlers and preschool‐aged children serve as a setting crucial to mental development, as stable relationships with caregivers and peers outside the family form. Children with early mental health disorders, especially externalising behaviours, are at increased risk of being excluded from access to these institutional services.

**Methods:**

The retrospective study investigates partial or full exclusion from childcare centre attendance among toddlers and preschoolers (*N* = 177) who received psychiatric day clinic treatment within two periods (2018/2019 and 2023/2024). We compared excluded and non‐excluded children regarding clinical parameters in a binary logistic regression. Furthermore, treatment trajectories were examined to assess reintegration into childcare settings as a potential therapeutic outcome.

**Results:**

Among the patients in the psychiatric day clinic for toddlers and preschoolers, the rate of exclusion from childcare centre attendance was 25.5% (2023/2024) and 8.0% (2018/2019), respectively. Boys were predominantly excluded from childcare centre attendance with 66.7% (2018/2019) and 92.3% (2023/2024). Overall for both cohorts, while male sex (*p* = 0.028) and cohort membership in the 2023/24 cohort (*p* = 0.009) predicted exclusion, other investigated variables (i.e., socioeconomic status, migration status, parental psychiatric condition) did not show an effect. In 78.1% of the excluded patients in the combined samples, (partial) reintegration or improvement of social integration was recorded or initiated during treatment.

**Conclusions:**

In our study, exclusion from childcare attendance differed between 2018/2019 and 2023/2024 in toddler and preschool children, especially affecting boys. In line with the UN Convention on the Rights of the Child, 1989, ensuring inclusive access to early education regardless of physical or mental impairments should be an important therapeutic goal. Mental health professionals should pay special attention to and actively support prevention and reintegration regarding early life exclusion experiences from childcare centres.

## Summary

- Children with behavioural disorders were disproportionately affected by exclusion from childcare centre attendance.
- Boys were more frequently affected by exclusion than girls.
- Exclusion rates were 8.0% (2018/2019) and 25.5% (2023/2024).
- Collaboration between psychiatric services and childcare centres may help to reduce exclusion.
- Supporting inclusion in childcare is an important task for mental health professionals.

**Abbreviations**
ADHD: attention‐deficit/hyperactivity disorder
CBCL: Child Behaviour Checklist
ICD: International Classification of Diseases
M: mean
n: number of cases
PSI: Parental Stress Index (EBI, German version of the PSI)
SD: standard deviation

## 1 Background

The attendance of a childcare centre plays a significant role in the socio‐emotional and cognitive development of children in the toddler and preschool‐aged range. Worldwide, approximately 91% of 3‐ to 5‐year‐olds are enrolled in childcare centres in high‐income countries (UNESCO. 2022). In particular, developing peer relationships and forming stable relationships with caregivers outside the family are important psychosocial developmental tasks at this stage (Groeneveld et al. 2016; Hay et al. 2004).

Developmental trajectories can be affected in the context of mental health disorders. In early childhood, these difficulties should be viewed from a biopsychosocial perspective, involving multidimensional factors such as the child's temperament and developing personality, parental influences and other developmental surrounding conditions (Calkins et al. 2013; Rettew and McKee 2005). According to a recent meta‐analysis, the prevalence of mental disorders in children under the age of seven is estimated to be 20.1% (Vasileva et al. 2021). In terms of symptomatology, a distinction can be made between internalising and externalising behavioural problems (Achenbach 1966).

In the context of such mental disorders, children are at increased risk of experiencing difficulties in their interaction with peers and caregivers. Based on the National Survey of Children's Health, young children with disabilities in the United States were found to be excluded at a rate of 5.4%, significantly higher than 1.2% observed among children without disabilities (Zeng et al. 2021).

These figures are particularly concerning in light of the Convention on the Rights of the Child by the United Nations, which affirms that children have a right to education and social participation, regardless of potential physical or mental impairments (United Nations 1989). Moreover, a study from the USA showed that children experiencing exclusion in childcare settings were more likely to be exposed to adverse experiences such as domestic violence, living in poverty, experiencing parental divorce or having a parent with mental illness or substance abuse (Zeng et al. 2019).

Given that parents of children with behavioural difficulties often themselves suffer from mental health problems (Lent et al. 2024; Nahar et al. 2025), an exclusion can thus contribute to further destabilisation of the family system. Moreover, parents are often already substantially burdened by their child's problematic behaviour and show symptoms of stress, which, in turn, exerts negative reciprocal effects on the child (Neece et al. 2012; Trumello et al. 2023). This dynamic is particularly important, as high levels of parental stress are recognised as a well‐established risk factor for dysfunctional parenting behaviour (Senn et al. 2023).

From a developmental perspective, the experience of exclusion from childcare centre attendance leads to simultaneous loss of relationships of both peers and secondary caregivers and therefore constitutes a considerable psychosocial stressor for the child. Additionally, resources to compensate stress and developmental risk in socio‐emotional and cognitive development in positive behavioural models might become less available. In this context of social exclusion, potential effects on the child's self‐esteem should be considered (Pilarz and Hill 2014; Rubin et al. 2009). Summing up, exclusion from childcare settings may contribute to considerable strain on the parent–child relationship through heightened parental stress, loss of resources and access to positive socio‐emotional, cognitive and behavioural models in dynamic interaction with other psychosocial stressors (Zinsser et al. 2024).

For clarity, in the present study, the term ‘parent’ refers to biological, adoptive or foster parents as well as legal guardians, whereas the term ‘caregiver’ refers to childcare providers and staff working in childcare centres.

Against this background, the current study aims to investigate exclusion experiences—including partial and full exclusions from childcare centres—among toddlers and preschool‐aged children served in a day clinic treatment setting. Investigation of exclusion patterns in this vulnerable group exhibiting behavioural difficulties is particularly important, given the increased risk of exclusion in the context of impairments as reported in current literature (Zeng et al. 2021). To our knowledge, there are no previous publications on child care exclusion rates in child and adolescent psychiatric treatment cohorts.

In particular, our study examines differences between children, (partially) excluded and non‐excluded from childcare centre attendance, regarding psychopathology, diagnostic classifications, sex distribution and parental stress levels in a retrospective approach. Furthermore, potential predictors of exclusion from childcare centre attendance are investigated. In addition, treatment trajectories are analysed to assess the extent to which reintegration into childcare centres can be achieved in the context of therapeutic intervention. Data were acquired from two treatment periods (2018/2019 and 2023/2024). Due to COVID‐19 pandemic‐related restrictions—in particular, the widespread closure of childcare centres between 2020 and 2022—data from that period were not suitable for evaluation. Thereby, this design also allows for the examination of potential changes over time.

## 2 Methods

A retrospective study was conducted analysing the records of patients who received treatment between 1 January 2018 until 31 December 2019 and 1 January 2023 until 31 December 2024 at the Psychiatric Day Clinic of the Department of Child and Adolescent Psychiatry at the University Hospital Muenster, Germany. Ethical approval for the retrospective data analysis was obtained by the local ethics committee (Reference Number: 2025‐420fs). The data were collected in Germany from a single psychiatric day clinic serving a defined regional catchment area. The clinic provides care for children with a broad range of psychiatric and developmental conditions. The study population predominantly consisted of children of German background, while also including children with migration backgrounds.

### 2.1 Description of the Psychiatric Day Clinic Treatment Setting

The study was conducted at a psychiatric day clinic for children aged 2 to 6 years with behavioural problems. The day clinic served as the source of the study sample and clinical data. Treatment was provided on either 2 or 3 days per week, allowing children to continue attending childcare centres on the remaining days. Consequently, all reported exclusion experiences referred to childcare centres and did not occur within the day clinic setting itself. The treatment structure also enabled close collaboration between the day clinic and childcare centres, which represent important developmental environments for the affected children.

### 2.2 Data Collection

Diagnoses were extracted according to the International Classification of Diseases (ICD‐10) (World Health Organization). In case of co‐occurring diagnoses, the leading diagnosis was used for further analyses. Individual diagnoses were subsequently grouped into predefined diagnostic categories based on ICD‐10 code ranges. These categories comprised emotional disorders with onset specific to childhood (F93.x), mixed disorders of conduct and emotions (F92.x), hyperkinetic disorders including ADHD (F90.x), conduct disorders (F91.x), autism spectrum disorders (F84.x) and disorders of social functioning with onset specific to childhood (F94.x). A detailed overview of all diagnostic categories and corresponding ICD‐10 codes is provided in Table 2.

Childcare status referred to the childcare centre attended by the child (regular setting, regular setting with inclusive support, or special needs setting) and the child's attendance status (regularly, partially excluded, frequent early pickups, fully excluded, or no childcare attendance).

Specifically, special needs settings were defined as facilities that explicitly accommodate children with special educational or developmental needs, whereas inclusive settings were defined as regular settings with integration support.

Exclusion referred exclusively to the childcare centre and not to the psychiatric day clinic. For cases in which an exclusion or partial exclusion was apparent, circumstances of reintegration into childcare settings during therapy (or if known after treatment) were additionally assessed.

Records of patients were reviewed for sociodemographic data (child's age at admission to the psychiatric day clinic, sex, single‐parent household/two‐parent household or foster care placement, socioeconomic burden, migration background and if documented mental problems of parents). Socioeconomic burden was defined by indicators such as unemployment and/or receipt of unemployment benefits, dependence on public welfare systems, reported financial problems or reported severe financial debt (not including loans within a covered loan plan). Migration background was defined as having at least one parent born outside of the country where the study was conducted.

Data were extracted retrospectively from electronic clinical records of patients treated in the psychiatric day clinic. The electronic records follow a standardised structural format with predefined sections (e.g., family history, developmental history, admission reports, progress notes and discharge summaries). However, the clinical information within these sections primarily consists of narrative free‐text documentation written by treating clinicians.

Information on exclusion from childcare attendance and reintegration into childcare settings was not available as predefined variables within the electronic record system. Therefore, these variables were identified through systematic manual review of relevant clinical documentation, including admission reports, progress notes, discharge summaries and documented communication with parents and childcare facilities.

Operational definitions were established prior to data extraction. Full exclusion was coded when documentation indicated that the child was no longer permitted to attend the childcare setting at all. Partial exclusion referred to situations in which attendance was restricted, for example through shortened care hours. Frequent early pickups referred to situations in which childcare staff repeatedly requested parents to collect the child before the scheduled end of the childcare day due to behavioural or emotional difficulties. Such situations were identified based on documentation in clinical notes or reports of communication with parents or childcare facilities. Reintegration was coded when documentation indicated that previously imposed restrictions on childcare attendance had been lifted and attendance time was increased again or planned or changes were initiated. This included both the return to a childcare centre following full exclusion and the gradual extension of attendance hours after a period of partial exclusion.

All variables were transferred into a structured dataset for statistical analysis.

For patients who underwent multiple treatment episodes, each episode was assessed for occurrences of childcare exclusion. Nevertheless, each patient was counted only once to avoid duplication in the overall case count.

### 2.3 Statistical Analysis

Data were recorded using Microsoft Excel 2010 (Microsoft Corporation, Redmond, WA, USA). SPSS Version 29.0 (IBM Corporation, Somers, NY, United States) was used for statistical analysis and for figures. Significance level was set at *p* < 0.05 for all tests (two‐sided).

Descriptive statistics were calculated to summarise sample characteristics, including age, sex and social background factors. Frequencies and percentages were used to report categorical variables (e.g., exclusion status, type of institution), whereas means and standard deviations were computed for continuous variables (e.g., age).

*N* = 8 patients in the 2018/19 cohort and *N* = 13 in the 2023/24 cohort had not yet attended a childcare centre and were therefore not eligible for the outcome of interest. These patients were excluded from subsequent analyses.

To examine factors associated with exclusion from childcare centre attendance, a binary logistic regression analysis was performed.

Assumptions of logistic regression were assessed. The dependent variable was binary, and observations were assumed to be independent. No relevant multicollinearity was detected (all VIFs < 1.2). Linearity of age in the logit was confirmed (*p* = 0.606), and no influential outliers were identified.

The dependent variable was exclusion status, coded as excluded and not excluded. Exclusion status was dichotomised into ‘any exclusion’ (including full, partial exclusion, frequent early pickups) and ‘no exclusion’.

Predictor variables were selected based on previous literature (Claussen et al. 2024; Gilliam 2005; Zeng et al. 2019) and included sex (reference category: male), age at admission to the psychiatric day clinic (continuous), migration background (reference: yes), socioeconomic burden (reference: yes) and parental psychiatric disease (reference: present).

To assess potential cohort effects, the cohort variable (2018/2019 vs. 2023/2024) was included as a predictor in the binary logistic regression model.

Diagnoses were not included as predictors but were reported on a descriptive level given that diagnostic categories in early childhood often show limited temporal stability, as symptom patterns may shift with age and developmental context as previously reported (Jobs et al. 2019). All potential predictor variables were entered simultaneously into the model using the standard entry method. Odds ratios (OR) and corresponding 95% confidence intervals (CI) were calculated for each predictor to assess the strength and direction of associations. The logistic regression model was evaluated using the omnibus test of model coefficients, Nagelkerke *R* ^2^ and overall classification accuracy.

No correction for multiple testing was applied, as predictors were defined a priori based on theoretical considerations.

Although only 3.77% of values were missing, listwise deletion in the multivariate analyses would have resulted in a disproportionately large loss of cases. To address this, single imputation (Zhang 2016) was performed using the standard SPSS procedure. Missing values were handled using single imputation in SPSS (linear regression imputation), in which missing values were estimated based on observed values of other variables included in the model. This approach was used to retain cases and avoid loss of statistical power. Sensitivity analyses indicated that the results remained significant in both the imputed and non‐imputed datasets as presented in the results section.

To assess the potential impact of missing data, additional regression analyses were conducted excluding variables with the highest proportion of missingness; results remained consistent with the primary analyses (socioeconomic status; parental mental disorder; migration status).

## 3 Results

### 3.1 Sociodemographic Data

#### 3.1.1 Sex Distribution

In the overall sample from 2018/2019, 63.9% of the patients were male (*n* = 82), whereas 36.1% were female (*n* = 33). In the overall sample from 2023/2024, 71.3% of the patients were male (*n* = 53), whereas 28.7% were female (*n* = 30).

Considering exclusions, 66.7% (*n* = 4) (2018/2019) and 92.3% (*n* = 24) (2023/2024) of the fully excluded or partially excluded patients were male. For detailed distribution regarding excluded and non‐excluded groups, see Table 1.

**TABLE 1:** Sociodemographic characteristics of the two investigated cohorts.

Characteristics	2018/2019 Cohort		2023/2024 Cohort	
	Not excluded group (*n* = 69)	Excluded group (*n* = 6)	Not excluded group (*n* = 76)	Excluded group (*n* = 26)
Age# in years, *M* ± *SD*	4.88 ± 1.08	5.46 ± 0.88	5.06 ± 1.06	5.17 ± 0.93
Duration of treatment in months, *M* ± *SD*	3.50 ± 1.54	4.35 ± 2.36	2.28 ± 1.23	2.24 ± 1.50
Sex, m/f, percent	66.7%	4/2	65.8%	92.3%
Parental mental disorder yes/no, percent	49.3%	5/1	40.8%/39.5%*	50.0%/34.6%*
Socioeconomic burden present, yes/no, percent	72.5%/24.6%*	5/1	69.7%/21.1%*	61.5%/23.1%*
Two‐parent/single‐parent/foster care placement, percent	60.9%/34.8%/4.3%	4/2/0	64.5%/32.9%/2.6%	53.8%/38.5%/7.7%
Joint/solo custody/guardian other than parents, percent	73.9%/20.3%/4.3%	4/2/0	86.8%/9.2%/2.6%	53.8%/38.5%/7.7%
Type of childcare (regular/special needs/inclusive setting)	59.4%/11.6%/26.1%	3/0/2	55.3%/7.9%/28.9%*	50.0%/15.4%/26.9%*
Migration background no/yes, percent	84.1%/15.9%	5/1	77.6%/18.4%	61.5%/26.9%*

*Note:* Mean values and standard deviations or frequencies presented as percentages are shown. #child's age at admission to the psychiatric day clinic, *M* = mean, SD = standard deviation. m = male, f = female. * = clinical records with missing data: parental mental disorder (*n* = 6, 3.4%), socioeconomic burden (*n* = 13, 7.3%), type of childcare (*n* = 4, 2.3%) and migration background (*n* = 6, 3.4%). For very small subgroups, results are presented as absolute numbers only.

#### 3.1.2 Age and Mean Duration of Treatment

The average age at admission to the psychiatric day clinic was 4.65 years (± 1.38) for the 2018/2019 cohort and 4.77 years (± 1.43) for the 2023/2024 cohort. The mean duration of treatment in the psychiatric day clinic setting was 3.51 ± 1.59 months in the 2018/2019 cohort and 2.23 ± 1.30 in the 2023/2024 cohort. For detailed information regarding excluded and non‐excluded groups, see Table 1.

#### 3.1.3 Psychosocial Circumstances

A significant proportion of investigated families of the overall population were affected by social stressors such as low socioeconomic status, single parenthood and parental mental health issues regardless of exclusion status. For detailed information, see Table 1.

### 3.2 Diagnostic Groups Across the Investigated Cohorts and Stratification for Childcare Centre Attendance

In the 2018/2019 cohort, children who were excluded from childcare attendance received the diagnoses of childhood emotional disorders (33.3%), mixed disorders of conduct and emotions (33.3%) and conduct disorders (33.3%). In the 2023/2024 cohort, the most common diagnoses among excluded children were childhood emotional disorders (42.3%), hyperkinetic disorders including ADHD (23.1%) and autism spectrum disorders (19.2%).

Among children in the not excluded group in 2018/2019, the most prevalent diagnoses were childhood emotional disorders (49.3%), mixed disorders of conduct and emotions (23.2%) and conduct disorders (11.6%). In the 2023/2024 not excluded group, childhood emotional disorders were most frequent (80.3%), followed by hyperkinetic disorders (6.6%) and autism spectrum disorders (3.9%). For detailed diagnostic frequencies and percentages, see Table 2.

**TABLE 2:** Diagnostic groups across the investigated cohorts and strata.

		Psychiatric day clinic cohorts			
		2018/2019		2023/2024	
		Childcare centre attendance			
Diagnostic group	ICD‐10‐Code	Not excluded (*n* = 69)	Excluded (*n* = 6)	Not excluded (*n* = 76)	Excluded (*n* = 26)
Emotional disorders with onset specific to childhood	*F93.x*	*N* = 34 49.3%	*N* = 2	*N* = 6 80.3%	*N* = 11 42.3%
Mixed disorders of conduct and emotions	*F92.x*	*N* = 16 23.2%	*N* = 2	*N* = 1	*N* = 2
Hyperkinetic disorders (including ADHD)	F90.x	*N* = 4 5.8%	*N* = 0	*N* = 5 6.6%	*N* = 6 23.1%
Conduct disorders	F91.x	*N* = 8 11.6%	*N* = 2	*N* = 3 3.9%	*N* = 1
Autism spectrum disorders	F84.x	*N* = 1	*N* = 0	*N* = 3 3.9%	*N* = 5 19.2%
Disorders of social functioning with onset specific to childhood	F94.x	*N* = 3 4.3%	*N* = 0	*N* = 1	*N* = 1

*Note:* Frequencies with percentages are shown for the several diagnostic groups according to the ICD‐10 classification (World Health Organization). Percentages are not reported for cells with very small counts (*n* ≤ 2).

### 3.3 Exclusion Rates

**FIGURE 1:**
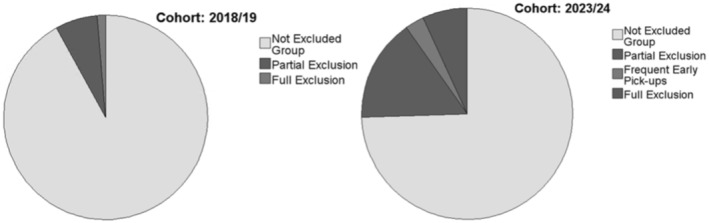
Attendance status and exclusion rates from childcare centre attendance in both treatment cohorts (2018/19 in %, 2023/24 in %). Not excluded group (92.0%/74.5%); partial exclusion (6.7%/15.7%); frequent early pickups (0.0%/2.9%); full exclusion (1.3%/6.9%).

In the two investigated cohorts, *n* = 83 (2018/2019) and *n* = 115 (2023/2024) patients were analysed. In the 2018/2019 cohort, a total of 75 children with planned childcare attendance were assessed, of whom six were excluded from childcare (excluded group). Sixty nine were enrolled regularly (non‐excluded group). Regarding the overall 2018/2019 treatment cohort, 7.23% of the patients had exclusion experiences. Regarding patients enrolled in childcare centres, 8.00% were affected by exclusion experiences. In the 2023/2024 cohort, a total of 102 children with planned childcare attendance were assessed, of whom 26 were excluded from childcare (excluded group). Seventy six were enrolled regularly (non‐excluded group). Among the overall 2023/2024 treatment cohort, 22.6% of patients experienced exclusion. When considering only those enrolled in childcare centres, the exclusion rate was 25.49%. For detailed information, see Figure 1.

### 3.4 Predictors of Exclusion

A binary logistic regression was conducted to identify potential predictors of exclusion (1 = excluded, 2 = not excluded). The independent variables were sex (reference: male), age (in years), parental psychiatric disorder (reference: present), socioeconomic burden (reference: yes), migration background (reference: yes) and cohort variable (reference: 2023/2024).

The overall model was statistically significant, *χ* ^2^(6) = 14.986, *p* = 0.009 and explained 19.0% of the variance in exclusion status (Nagelkerke *R* ^*2*^ = [0.190]). It correctly classified 84.2% of cases.

Male sex was associated with significantly higher odds of exclusion compared with female sex (OR = 3.591, 95% CI [1.145, 11.264], *p* = 0.028). Cohort membership (2023/2024 vs. 2018/2019) was associated with significantly higher odds of exclusion compared with the 2018/2019 cohort (OR = 3.653, 95% CI [1.386, 9.628], *p* = 0.009).

Age at admission to the psychiatric day clinic, socioeconomic burden, migration background and parental psychiatric disorder were no statistically significant predictors of exclusion (*p* > 0.05).

A complete‐case analysis including only participants with no missing data was conducted to assess the robustness of the findings. The results were consistent with the primary analysis. Male sex was associated with significantly higher odds of exclusion (OR = 5.281, 95% CI [1.120, 24.897], *p* = 0.035), as was cohort membership (2023/2024 vs. 2018/2019; OR = 3.309, 95% CI [1.181, 9.268], *p* = 0.023). Age at admission, parental psychiatric disorder, socioeconomic burden and migration background were not significantly associated with exclusion (all *p* > 0.05).

Additional analyses using multiple imputation yielded comparable results. Male sex (OR = 3.698, *p* = 0.026) and cohort membership (OR = 3.690, *p* = 0.008) remained significant predictors, whereas migration background (OR = 1.689, *p* = 0.317), parental psychiatric disorder (OR = 0.545, *p* = 0.222), socioeconomic burden (OR = 1.452, *p* = 0.506) and age (OR = 1.315, *p* = 0.224) were not significantly associated with exclusion.

Sensitivity and specificity were calculated, yielding a specificity of 97.9% and a sensitivity of 9.4%; the base rate of exclusion in the sample was 18%.

### 3.5 Reintegration During Treatment

In the combined sample of both cohorts, 78.1% of children experienced either fully implemented or at least partially initiated steps toward reintegration or improved social participation.

In the 2018/2019 cohort, 83.3% of excluded children showed improvements in social integration during treatment, such as extended care hours or better inclusion in childcare settings.

Similarly, in the 2023/2024 cohort, 76.9% of excluded children benefited from reintegration efforts. Specifically, in 42.3% of cases, concrete improvements in social integration were implemented (e.g., extended care hours or better childcare integration), whereas in 34.6%, action plans were collaboratively developed with childcare centres and families (e.g., planned transitions, increased support hours, or integration aides). See Table 3 for a detailed description of reintegration efforts.

**TABLE 3:** Outcomes of reintegration efforts across both cohorts.

Category	Description	Examples from the data	*n*	Percent
Improved social integration	Concrete functional improvements in daycare participation and social functioning	Extended care hours; improved integration into childcare routines; increased attendance; support via integration aide; improved peer interaction within the childcare setting (e.g., participation in group activities, forming peer contacts); reported symptom improvement by childcare staff	13	42.3
Intervention planning/initiation of reintegration measures	Development or initiation of structured interventions in collaboration with childcare centres and families	Stepwise reintegration plans; recommendations (special education daycare, integration aide, additional support services); coordination with childcare; planned transitions	11	34.6
No or unclear benefit	No documented improvement or insufficient information	No extension of care hours, missing data	8	23.1

*Note:* Displayed are frequencies and percentages of outcomes following reintegration efforts across both cohorts. Categories were coded such that cases were assigned hierarchically to either (1) improved social integration, (2) intervention planning/Initiation of reintegration measures or (3) no or unclear benefit. Percentages refer to the total sample of excluded children (*N* = 32).

## 4 Discussion

Our retrospective study investigated experiences of exclusion from childcare settings among toddlers and preschoolers in a day clinic setting. We compared excluded and non‐excluded children regarding psychopathology, diagnoses, sex, parental stress and treatment outcomes, including reintegration into childcare centres. Our analyses yielded the following main results: The analysis of treatment data from our psychiatric day clinic during the years 2023/2024 showed that 25.5% of patients were particularly affected by childcare exclusions, which represents an increase compared with the data of 8.0% recorded in 2018/2019. Cohort membership in 2023/2024 significantly predicted exclusion when compared with membership in the 2018/2019 cohort.

In both samples, boys were especially excluded from childcare (*p* = 0.028), comprising 66.7% (*n* = 4) (2018/2019) and 92.3% (*n* = 24) (2023/2024) of the excluded children. Other potential risk factors related to exclusion, such as age, parental psychiatric disorders, high socioeconomic burden and migration background (Zeng et al. 2019) were no predictors of exclusion. In 78.1% of the cases, recorded or initiated (partial) reintegration or improvement of social integration was seen.

Our results align with a particularly concerning trend that has been observed in toddlers and preschoolers, where expulsion rates are reported to be three times higher than those among school‐aged children, according to the US National Prekindergarten Study (Gilliam 2005). These findings need to be considered in light of well‐documented detrimental long‐term effects on children's educational trajectories, social participation and adult educational attainment (Meek and Gilliam 2016). Our finding of boys being disproportionately often affected by exclusions in childcare is in line with previous literature (Claussen et al. 2024; Gilliam 2005). However, this might be associated with the fact that, at least in our psychiatric day clinic, most preschool‐aged patients are boys. Moreover, this result might be explained by the issue that the distribution of internalising and externalising disorders in pre‐school‐aged patients is related to sex (Klein et al. 2013; Wichstrøm et al. 2012). Nonetheless, these findings should be interpreted with caution given the small and imbalanced sample sizes in the excluded subgroups, particularly in the 2018/2019 cohort, which may limit the stability and generalisability of the observed associations.

In the current study, we observed a pronounced temporal increase in exclusion, with markedly higher rates in 2023/2024 compared with 2018/2019. Cohort membership in 2023/2024 significantly predicted exclusion in the regression model. These results may indicate that exclusion has risen over time. Our results further raise the question of whether childcare centres may be insufficiently equipped to manage children with significant behavioural challenges. A broader shortage of specialised staff and a lack of resources to support this vulnerable group should be considered. Problematic behaviour in children may lead to increased stress among caregivers or exacerbate existing burdens (Stein et al. 2022). Additionally, caregiver‐related factors such as attributions and biases related to the child should be considered (Newland et al. 2024).

Moreover, caregiver–parent interactions appear to play an important role in the development and maintenance of exclusion processes (Zulauf and Zinsser 2019). In the context of our findings, this suggests that not only child‐related factors but also the broader interactional context may contribute to exclusion decisions. Challenging child behaviour may increase caregiver stress and strain communication with parents, potentially leading to escalating dynamics that culminate in exclusion. Therefore, structured support for both caregivers and parents—particularly focusing on shared understanding and consistent strategies in managing challenging behaviours—may represent a key mechanism for preventing exclusion (Gilliam 2005; Hoover et al. 2012). Nevertheless, due to the limited sample size, this temporal trend should be interpreted cautiously, as it may also reflect sample‐specific variation rather than a generalisable shift over time.

The observed increase in exclusion rates in the 2023/2024 cohort should be interpreted in the context of the COVID‐19 pandemic and its potential impact on early childhood development. Children in this cohort were exposed to pandemic‐related disruptions during critical developmental periods, including reduced access to childcare environments, limited peer interaction and increased exposure to family‐related stress. Recent studies indicate that the COVID‐19 pandemic has been associated with increases in emotional and behavioural problems in young children, as well as impairments in social–emotional development (Alcon et al. 2024).

The differences in exclusion rates between the two cohorts should be interpreted with caution, as several alternative explanations need to be considered. System‐level factors may have contributed to the observed pattern. These include changes in referral pathways, increased awareness and identification of behavioural difficulties and shifts in diagnostic practices over time. Furthermore, post‐pandemic pressures on childcare systems and child and adolescent mental health services may have influenced both access to care and thresholds for referral and exclusion.

It is therefore possible that the higher exclusion rates observed in the 2023/2024 cohort reflect, at least in part, changes in service utilisation and detection rather than a true increase in exclusion at the population level. Accordingly, the present findings should be interpreted as differences between cohorts within a clinical sample, rather than as evidence of a definitive temporal increase in exclusion rates. From a clinical perspective, Claussen et al. consider preschool expulsion as an indicator of elevated risk: Among children diagnosed with ADHD, investigated in the US National Survey of Children's Health (2016–2020), a history of preschool expulsion was associated with more severe ADHD symptoms, co‐occurring behavioural and developmental disorders, earlier diagnosis and medication initiation, as well as academic and social impairment (Claussen et al. 2024). These results can be discussed in the context of our results of 19.2% of the excluded patients of the 2023/2024 cohort who were affected by hyperkinetic disorders (including ADHD). An additional factor that may have contributed to the higher rate of ADHD diagnoses in the second cohort is the observed difference in age distribution. Children in the 2023/2024 cohort were, on average, older than those in the 2018/2019 cohort. This is relevant because the diagnosis of ADHD requires the presence of developmentally inappropriate and persistent patterns of inattention and/or hyperactivity (American Psychiatric Association and Association 2013), which can be difficult to reliably assess in very young children. The observed increase in ADHD diagnoses in the second cohort may thus be at least partially attributable to the moderate higher mean age.

As with all decisions related to child development, the child's well‐being and the best interests of the child should always be the first priority (United Nations 1989). Therefore, given the legal right to education and participation and the dramatic effects of exclusion, it is essential to intervene before exclusion decisions are made. In line with our findings, an important task for mental health professionals is to initiate collaboration with childcare, in agreement with the affected families, to identify and prevent exclusion dynamics at an early stage (Gilliam 2005; Hoover et al. 2012). Moreover, reintegration into childcare settings is an important topic to be addressed in treatment.

In summary, exclusion from childcare should be viewed as a significant intervention with the potential to negatively affect the development of the young patients and lead to heightened family stress (Giordano et al. 2025; Williams et al. 2023). Caregivers in childcare centres should receive adequate support and training to reduce trajectories of exclusion. Additionally, mental health professionals in child and adolescent psychiatry play a pivotal role in supporting the inclusion of children in early childhood education settings by actively counteracting processes of social exclusion.

### 4.1 Strengths and Limitations

The main strength of the manuscript is its focus on examining a vulnerable target group of children with mental disorders at an early age. In this context, particular emphasis is placed on early childhood education settings as a critical learning and social environment for early development of the affected children.

On the other hand, our study shows some limitations. Firstly, the retrospective design limits causal interpretations. Secondly, missing data in some clinical parameters and questionnaires may have biased the results. Finally, although ICD‐10 diagnoses were available, no diagnosis‐specific or behaviour‐specific information could be included in the statistical model. This decision was made because the relatively small sample size did not allow for meaningful stratification across numerous diagnostic categories, and because psychiatric diagnoses in preschool‐aged children are limited in temporal stability (Jobs et al. 2019).

The base rate of exclusion in the sample was relatively low (approximately 18%), indicating an imbalance in outcome distribution. Accordingly, overall classification accuracy should be interpreted with caution, as it is largely driven by correct classification of the majority class. Consistent with this, the model showed high specificity but very low sensitivity. Nevertheless, significant associations between key predictors (e.g., sex and cohort membership) and exclusion status were observed, supporting the relevance of these factors in this clinical sample.

A further limitation is that childcare settings were categorised into regular, inclusive and special needs settings, but no more detailed differentiation regarding specific pedagogical approaches or care structures was available. Even within these categories, substantial heterogeneity exists, which may have influenced children's behavioural outcomes and the likelihood of exclusion.

Additionally, as a relevant limitation, it has to be stated that no long‐term follow‐up data on developmental and clinical outcomes after exclusion were available. Due to the small number of full exclusion cases (*n* = 8), differentiation between full and partial exclusion was not feasible; future studies with larger samples may enable more detailed stratified analyses.

## 5 Conclusion

Early behavioural disorders can contribute to exclusion from childcare centres, thereby reinforcing social disadvantage at a critical developmental stage. In our treatment samples, exclusion rates differed substantially between cohorts, with 8.0% of the 2018/2019 cohort compared with 25.5% in the 2023/2024 cohort. In line with this descriptive increase, cohort membership in 2023/2024 significantly predicted exclusion in the regression model. However, these findings should be interpreted with caution. The observed differences may not necessarily reflect a true temporal increase in exclusion rates but could also be influenced by changes in referral patterns, diagnostic practices or service‐related factors between cohorts.

To our knowledge, this is the first study to investigate exclusion rates from childcare in a treatment sample from a child and adolescent mental health unit. In line with current literature investigating general population samples or childcare samples, patterns of exclusions in toddler and preschool‐aged children were apparent especially in boys and in the context of behavioural problems (Claussen et al. 2024; Gilliam 2005). The significance of our findings lies in the high potential of therapeutic intervention and collaboration with the childcare centres, which is shown in 78.12% of the cases with recorded or initiated (partial) reintegration or improvement of social integration. Summing up, increasing awareness and establishing therapeutic collaboration between mental health professionals and caregivers in childcare centres in agreement with families appear to be highly relevant to reduce exclusion experiences for the young patients. Future studies should focus on the vulnerable patient age group of toddlers and preschool children to understand and reduce exclusion trajectories in early life.

## Author Contributions

**Linda Meredith Bonnekoh:** data curation, conceptualization, investigation, formal analysis, funding acquisition, visualization, writing – original draft, writing – review and editing. **Lennart Friese:** data curation, formal analysis, writing – review and editing. **Ruth Helena Fellmeth:** data curation, writing – original draft. **Héctor Andrés Sánchez Guerrero:** writing – original draft. **Selina Hansen:** data curation, writing – original draft. **Jörg Michael Müller:** methodology, writing – review and editing. **Julia Hubbert:** methodology, writing – review and editing. **Udo Dannlowski:** resources, writing – review and editing. **Georg Romer:** writing – review and editing, project administration, resources. **Marius Janßen:** conceptualization, supervision, writing – review and editing.

## Funding

This work was supported in part by the ‘Innovative Medizinische Forschung’ (IMF) by the medical faculty of the University of Münster awarded to LMB (ME122205).

## Ethics Statement

The retrospective data analysis was performed in accordance with the Declaration of Helsinki and was reviewed and approved by the local Ethics Committee of the Ethikkommission Westfalen‐Lippe, Germany (Reference Number: 2025‐420fs).

## Consent

Not applicable (retrospective design, approved by the local Ethics Committee).

## Conflicts of Interest

The authors declare no conflicts of interest.

## Permission to Reproduce Material

No previously published material has been reproduced in this manuscript.

## Data Availability

The datasets generated and analysed during the current study are not publicly available due to the legal restrictions and ethical implications (retrospective analysis, smaller sample sizes).
